# Ticagrelor plus aspirin in patients with minor ischemic stroke and transient ischemic attack: a network meta-analysis

**DOI:** 10.1186/s12883-023-03356-7

**Published:** 2023-08-14

**Authors:** Mingxia Li, Qianru Yang, Jiankuan Shi, Xiaolong Zhang, Hong Lin, Fangfang Ge

**Affiliations:** Department of Neurology, Xi’an International Medical Center Hospital, Xitai Road, Gaoxin District, Xi’an City, 710010 Shaanxi Province China

**Keywords:** Ischemic stroke, Transient ischemic attack, Antiplatelet, Ticagrelor, Meta-analysis

## Abstract

**Background:**

Dual antiplatelet therapy (DAPT) with aspirin and clopidogrel was recommended as the secondary prevention of minor ischemic stroke or transient ischaemic attack (TIA). However, genetic polymorphisms of CYP2C19 had been identified as the major cause of poor responsiveness to clopidogrel. Ticagrelor, unlike clopidogrel, did not depend on metabolic activation, but it remained unclear whether ticagrelor was superior to clopidogrel in ischemic stroke. We performed a network meta-analysis to compare the efficacy and safety of ticagrelor, clopidogrel, and aspirin in the minor ischemic stroke and TIA populations.

**Methods:**

Databases of Cochrane Library, ClinicalTrials.gov, and PubMed were searched up to June 19, 2023. Randomized controlled trials (RCTs) assessing antiplatelet drugs for minor stroke or TIA were included. Statistical processing was conducted by using multivariate meta-analysis routines of STATA.

**Results:**

Seven RCTs were included involving 41,745 participants. There was no significant difference between the two DAPTs in preventing stroke recurrence (OR, 1.16; 95% CI, 0.93-1.44), ischemic stroke recurrence (OR, 1.16; 95% CI, 0.93-1.45), and major hemorrhage (OR, 1.22; 95% CI, 0.62,2.39). Compared with aspirin alone, the two DAPT regimen reduced the risk of stroke recurrence (clopidogrel: OR, 0.69; 95% CI, 0.60-0.80, ticagrelor: OR, 0.66; 95% CI, 0.49-0.87) and ischemic stroke recurrence, but increased the incidence of major hemorrhage (clopidogrel: OR, 2.05; 95% CI, 1.22- 3.77; ticagrelor: OR, 2.55; 95% CI, 1.25-4.99). Despite being associated with a higher risk of any bleeding, ticagrelor did not impact the composite of vascular events or mortality. While ticagrelor and aspirin reduced the risk of ischemic stroke recurrence (OR, 0.77; 95% CI, 0.63- 0.92) without increasing the risk of major bleeding (OR 0.94; 95% CI 0.45–1.95) in the Asian population mainly Chinese.

**Conclusions:**

DAPT was superior to aspirin in stroke prevention, but little difference existed between the two DAPT regimens. Asian population mainly Chinese may benefit from DAPT with aspirin and ticagrelor. But further head-to-head RCTs are needed to validate the study results.

**Supplementary Information:**

The online version contains supplementary material available at 10.1186/s12883-023-03356-7.

## Introduction

The risk of recurrent stroke ranges 10% to 20% in the 90 days after a minor ischemic stroke or a transient ischemic attack (TIA) with the index event [1–4]. Several randomized controlled trials (RCTs) have confirmed the superior effectiveness of dual antiplatelet therapy (DAPT) with clopidogrel and aspirin over aspirin alone in reducing subsequent events in patients with minor stroke or TIA [5–7].

However, clopidogrel resistance occurrs in 5% to 60% of patients. Genetic polymorphisms of CYP2C19 have been identified as the major cause of poor responsiveness to clopidogrel [8, 9]. Compared with the white ethnic group, the Asian race is associated with a higher likelihood of CYP2C19 carriers [10, 11], and hence clopidogrel might be less effective for secondary stroke prevention in Asian populations.

Ticagrelor is a reversible P2Y12 receptor antagonist, which unlike clopidogrel, does not require conversion from prodrug to active drug in the liver. Previous studies had shown that ticagrelor was more effective than clopidogrel in inhibiting platelet reactivity and reducing the recurrence of ischemic vascular events in patients with acute coronary syndrome [12].

Despite numerous trial studies, it remains unclear whether ticagrelor is superior to clopidogrel in ischemic stroke. SOCRATES trial [13] demonstrated comparable outcomes between ticagrelor and aspirin in reducing the rate of stroke, myocardial infarction, or death at 90 days. Furthermore, DAPT with ticagrelor and aspirin exhibited incremental benefits over aspirin monotherapy in the THALES trial, while severe bleeding was more frequent with ticagrelor [14]. RCTs directly comparing the two DAPT regimens were evaluated in PRINCE [15] and CHANCE-2 [16], while there was no consistent result on aspirin with ticagrelor in secondary prevention of in minor ischemic stroke or high-risk TIA. The previous meta analysis did not include theCHANCE-2 study which compared the two DAPT regimes [17],the other only included the two Chinese trials pooled analysis [18].

In summary, the existing studies are limited to the role of aspirin and ticagrelor in stroke prevention. We performed a systematic review and network meta-analysis (NMA) to synthesize available evidence and compare the relative efficacy and safety of ticagrelor, clopidogrel and aspirin in the acute minor ischemic stroke and TIA populations.

## Methods

The NMA was conducted in accordance with the preferred reporting items for systematic review and meta-analysis (PRISMA) guidelines [19]. The study did not require any ethics committee approval because of its non-experimental design.

### Search strategy

We searched Cochrane Library, ClinicalTrials.gov,and PubMed databases (published until Jun 19, 2023) with keywords ‘antiplatelet therapy’, ‘aspirin’, ‘clopidogrel’, ‘ticagrelor’, ‘minor stroke’, ‘transient ischemic attack’ and ‘randomized controlled trial’. The search was restricted to the English language and published articles.

### Study selection

Two authors (SJK, ZXL) screened the search results, excluded irrelevant publications based on the title and abstract, and then obtained full texts of potentially relevant articles. The other two authors (YQR, LMX) selected eligible studies according to the following criteria: (i) participants with a National Institutes of Health Stroke Scale (NIHSS) score of 5 or less for ischemic stroke or an ABCD2 score of 4 or higher for TIA (ii) DAPT or monotherapy was initiated within 48 h after the index event (iii); only randomized controlled trials were included; (iv)the efficacy outcomes were evaluated as stroke recurrence, ischemic stroke recurrence and major vascular events including stroke, myocardial infarction,and vascular death; the safety outcomes were evaluated as major bleeding,any bleeding and mortality. Bleeding events were classified by the PLATO bleeding definition. In addition, trials were excluded based on the following criteria: (i) those with follow-up averaging < 30 days; (ii) published only as abstracts; (iii) Use of antiplatelet agents other than Clopidogrel, Ticagrelor, or Aspirin. (iv) pilot study.

### Data extraction

Two neurologists (LMX,GFF) independently extracted the following information: data on methodological features, sample size of patients treated, study centers (single or multicenter), targeted population, study design, treatment groups (medications and dosages), onset-to-treatment interval, treatment duration, duration of follow-up and the number of all efficacy and safety events.

### Transitivity, risk of bias, and quality assessment

Population and study design was analyzed to check for transitivity. Age, sex, follow-up, treatment duration and severity of stroke were pre-specified as relevant factors that could potentially affect the transitivity assumption.

We assessed the risk of bias in included RCTs using the Cochrane Risk of Bias tool for randomized trials version 2(RoB2) [20], the efficacy outcome of stroke recurrence was assessed for risk of bias. Five parameters (the randomization process, intended interventions, missing outcome data, measurement of the outcome and selection of the reported result) were rated either as low risk, of some concern, or high risk.

The quality of evidence for each outcome subjected to network meta-analysis was rated and an overall grading of the quality of evidence was produced, with reference to the grading of recommendations assessment, development and evaluation (GRADE) system [21] with CINeMA software [22].

### Data synthesis and analysis

The NMA pooled all the direct and indirect evidence and compared several treatments simultaneously by using the network relationship. In the NMA, the OR and 95% confidence interval were applied for the efficacy and safety evaluation of various treatments. Statistical processing was conducted by using multivariate meta-analysis routines of STATA version 14.2 using metwork package. In addition, we also calculated secondary measures of treatment effect for each intervention in the form of surface under the cumulative rank curve (SUCRA) probabilities and treatment rankings. The SUCRA value varies from 0 to 100%, with a higher SUCRA value indicating a better treatment outcome for efficacy outcomes and lower risk for safety outcomes. We also compared the results of direct, indirect, and network evidence to evaluate the consistency of every outcome between direct and indirect evidence. If there was inconsistency, the inconsistency model was used for analysis. We performed a sensitivity analysis by excluding one study that was deemed to be heterogeneous. We assessed the statistical between-study heterogeneity as the *I*^2^ statistic (%).A high degree of heterogeneity existed between studies when the *I*^2^ statistic was greater than 50%, we performed a pairwise meta-analysis using a random-effects model among the included studies. Publication bias was assessed by visual examination of funnel plots.

## Results

**Fig. 1:**
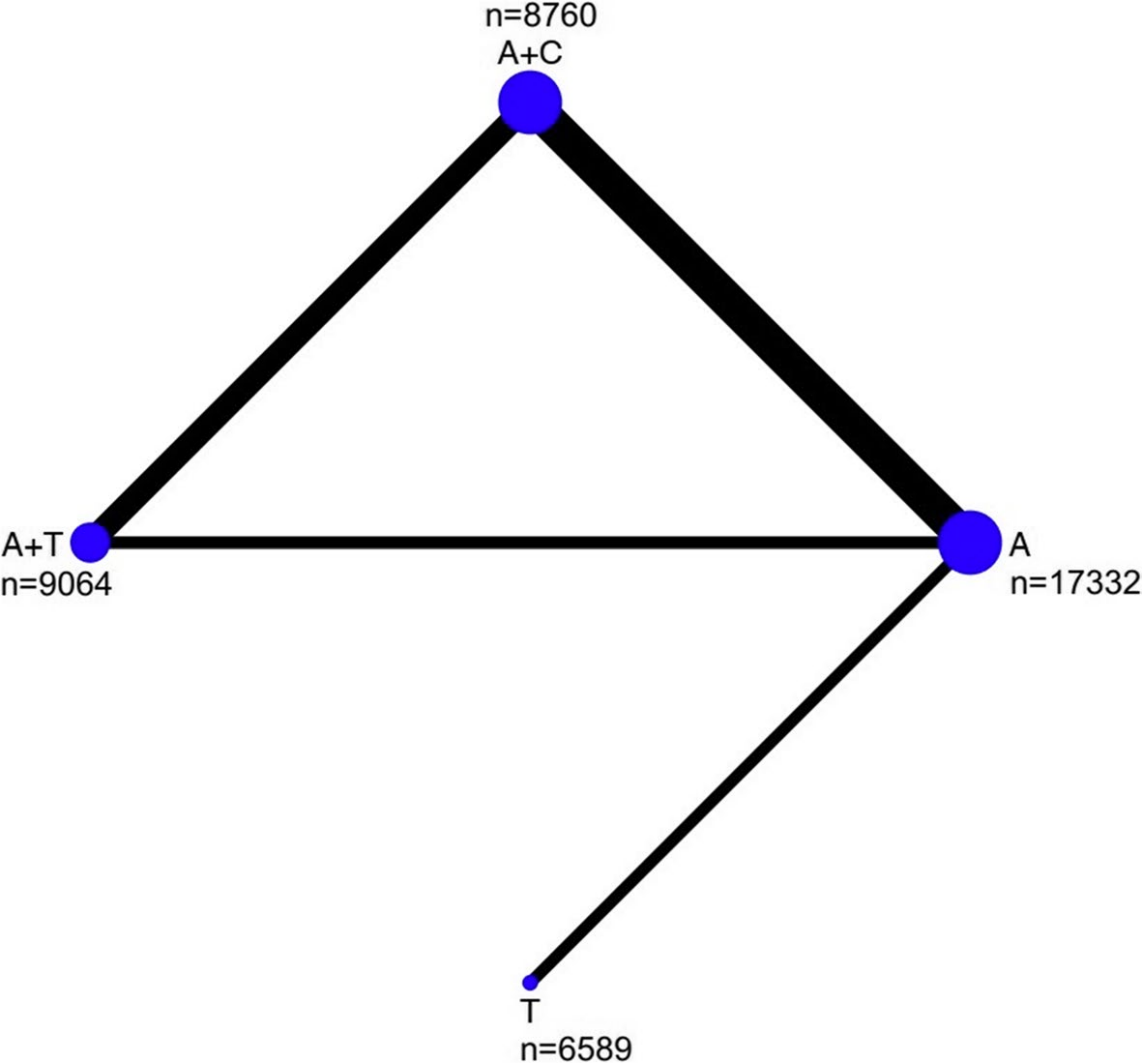
Network diagram of the total number of patients analyzed in each treatment arm

Seven RCTs [5–7, 13–16] were included in our NMA with a total of 41,745 participants enrolled. The search procedure was listed in Figure S1. Among the study patients, 17,332 received aspirin, 8,760 received clopidogrel and aspirin, 9,064 received ticagrelor and aspirin, and the remaining 6,589 received ticagrelor alone. The mean age of patients was 65 years, and the follow-up ranged from 30 to 90 days. The treatment window was less than 24 h. Within the included studies, three RCTs compared clopidogrel-led DAPT against aspirin, one compared ticagrelor-led DAPT with aspirin, and another benchmarked ticagrelor alone against aspirin. The other two RCTs performed a head-to-head comparison between the two DAPT regimens. The numbers of participants ranged from 392 to 13,199 in these respective studies. Transitivity can be clinically assumed because of similar baseline characteristics and potential treatment effect modifiers.All studies included in the current NMA were judged to have a low risk of bias in all RoB2 domains (Figure S2).Detailed patient characteristics were listed in Table 1 and the corresponding network diagram was presented in Fig. 1. Table 2 summarized random effect model measures for efficacy and safety outcome.

**Table 1:** Characteristics of studies included in the meta-analysis

Study	teatment vs control	Loading dose	country	total	Female	treatment window form onset	treatment duration	Follow-up	Median age	Asian	NIHSS	ABCD2	Lost
CHANCE-2 2021 [16]	A + T vs A + C(A100mg,T90mg bid,C75mg	T180mg,A300mg,C300mg	China	6412	2170	24 h	21d	90d	UNK	6412	≤ 3	≥ 4	0
THALES 2020 [14]	A + T vs A (T90 bid,A75-100 mg)	T180mg,A300-325 mg)	28 countries	11,016	4279	24 h	30d	30d	65	4729	≤ 5	≥ 6	UNK
PRINCE 2019 [15]	A + T vs A + C(A100mg,T90mg bid,C75mg	T180mg,A100-300 mg,C300mg	China	675	181	24 h	21d	90d	61	675	≤ 3	≥ 4	12
POINT 2018 [6]	A + C vs A(C 75 mg, A50-325 mg)	C 600 mg		4881	2195	12 h	90d	90d	65	144	≤ 3	≥ 4	199
SOCRATES 2016 [13]	T vs A (T90bid,A100mg)	T180mg,A300mg	33countries	13,199	5483	24 h	90d	90d	66	3906	≤ 5	≥ 4	2
CHANCE 2013 [5]	A + C vs A(C 75 mg, A50-325 mg)	C300mg	China	5170	1750	24 h	21d	90d	63	5170	≤ 3	≥ 4	36
FASTER 2007 [7]	A + C vs A(C 75 mg, A81mg)	C300mg A162mg	Canada	392	UNK	24 h	90d	90d	UNK	0	≤ 3	NA	UNK

*A* aspirin, *C* clopidogrel, *T* ticagrelor, *h* hour, *d* day, *UNK* unknown, *NIHSS* National Institutes of Health Stroke Scale

**Table 2:** The random effect model measures for efficacy and safety outcome and sensitivity analysis

		OR (95% CI)				SUCRA			
Primary Analysis	Outcome measure	A + C vs A	A + T vs A	A + T vs A + C	T vs A	A + T	A + C	A	T
	stroke	0.69 (0.60,0.80)	0.66 (0.49,0.87)	1.16 (0.93,1.44)	0.80 (0.68,0.94)	61.00%	96.20%	0.70%	42.10%
	ischemic stroke	0.68 (0.58,0.78)	0.66 (0.49,0.88)	1.16 (0.93,1.45)	0.78 (0.67,0.92)	63.60%	96.50%	0.90%	39%
	vascular event	0.97 (0.63,1.49)	0.71 (0.36,1.40)	0.73 (0.43,1.23)	0.88 (0.46,1.70)	80.80%	36%	31.90%	51.40%
	major bleeding	2.05 (1.11,3.77)	2.50 (1.25,4.99)	1.22 (0.62,2.39)	0.82 (0.40,1.68)	9.90%	25.40%	76.10%	88.60%
	any bleeding	2.10 (1.22,3.59)	4.14 (2.00,8.55)	1.97 (1.21,3.22)	1.30 (0.66,2.55)	0.40%	38.00%	92.70%	68.90%
	mortality	1.50 (0.93,2.41)	1.17 (0.76,1.82)	0.78 (0.46,1.32)	1.18 (0.83,1.67)	52.10%	14.30%	84.20%	49.30%
Sensitivity Analysis	ischemic stroke	0.70 (0.60,0.80)	0.76(0.65,0.88)	1.08(0.88,1.33)		70.60%	92.10%	0.80%	36.50%
	major bleeding	1.83 (0.99,3.40)	3.0 (1.44,6.25)	1.64 (0.70,3.82)		4.30%	31.30%	74.90%	89.40%

*A* aspirin, *C* clopidogrel, *T* ticagrelor

### Stroke

**Fig. 2:**
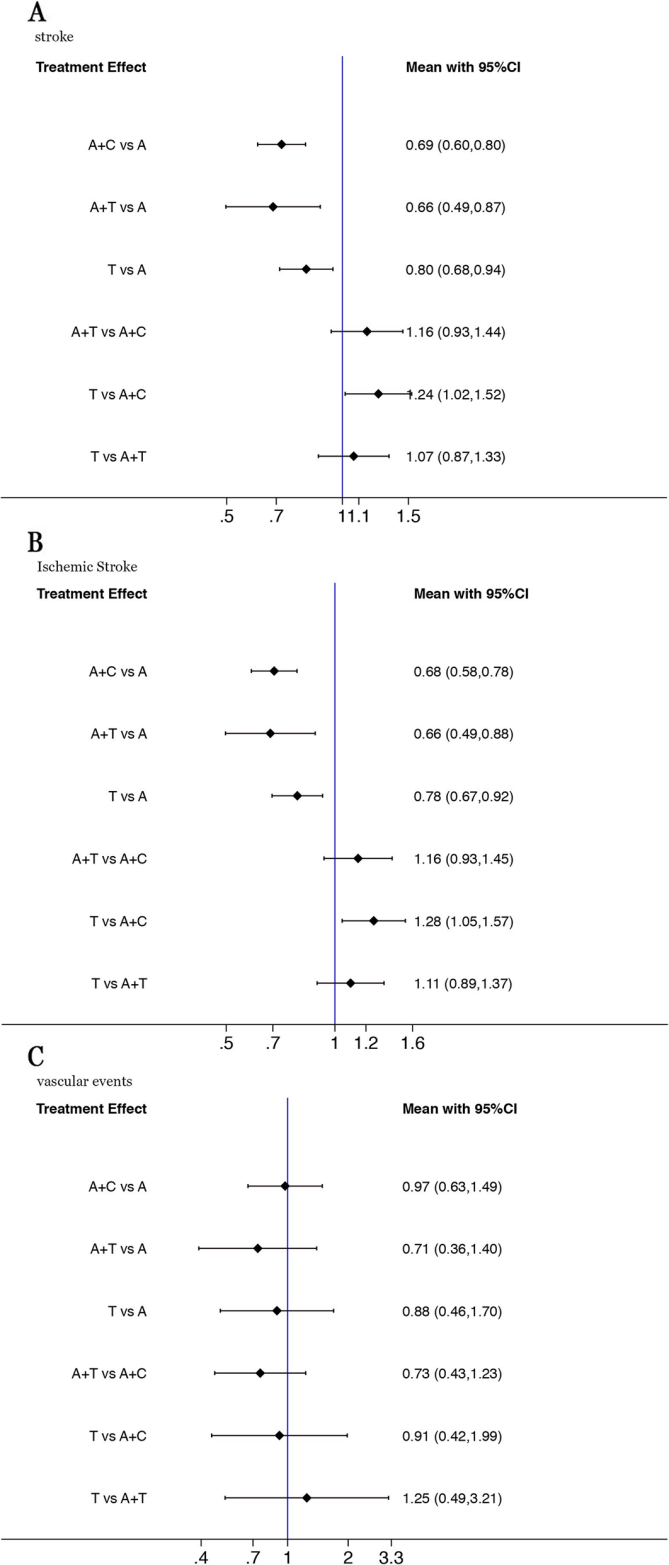
Forest plot for the efficacy outcomes between treatment vs control (**A**: stroke, **B**: ischemic stroke, **C**: vascular events)

The evidence network for the stroke analysis was comprised of 7 RCTs with 41,745 participants.The incidence of stroke recurrence did not differ significantly in ticagrelor and aspirin versus clopidogrel and aspirin (OR, 1.16; 95% CI, 0.93–1.44). DAPT with Clopidogrel-aspirin or ticagrelor -aspirin was both more efficacious compared with aspirin alone (clopidogrel: OR, 0.69; 95% CI, 0.60–0.80, ticagrelor: OR, 0.66; 95% CI, 0.49–0.87). ticagrelor was more efficacious compared with aspirin (OR, 0.80; 95% CI, 0.68–0.94) (Fig. 2A). Low evidence recommendations as heterogeneity and inconsistency. Clopidogrel and aspirin had the highest SUCRA value for stroke recurrence at 96.2%, followed by ticagrelor and aspirin (SUCRA, 61.0%), and ticagrelor and aspirin alone (SUCRA, 42.1%and 0.7%;) (Figure S3A).

**Fig. 3:**
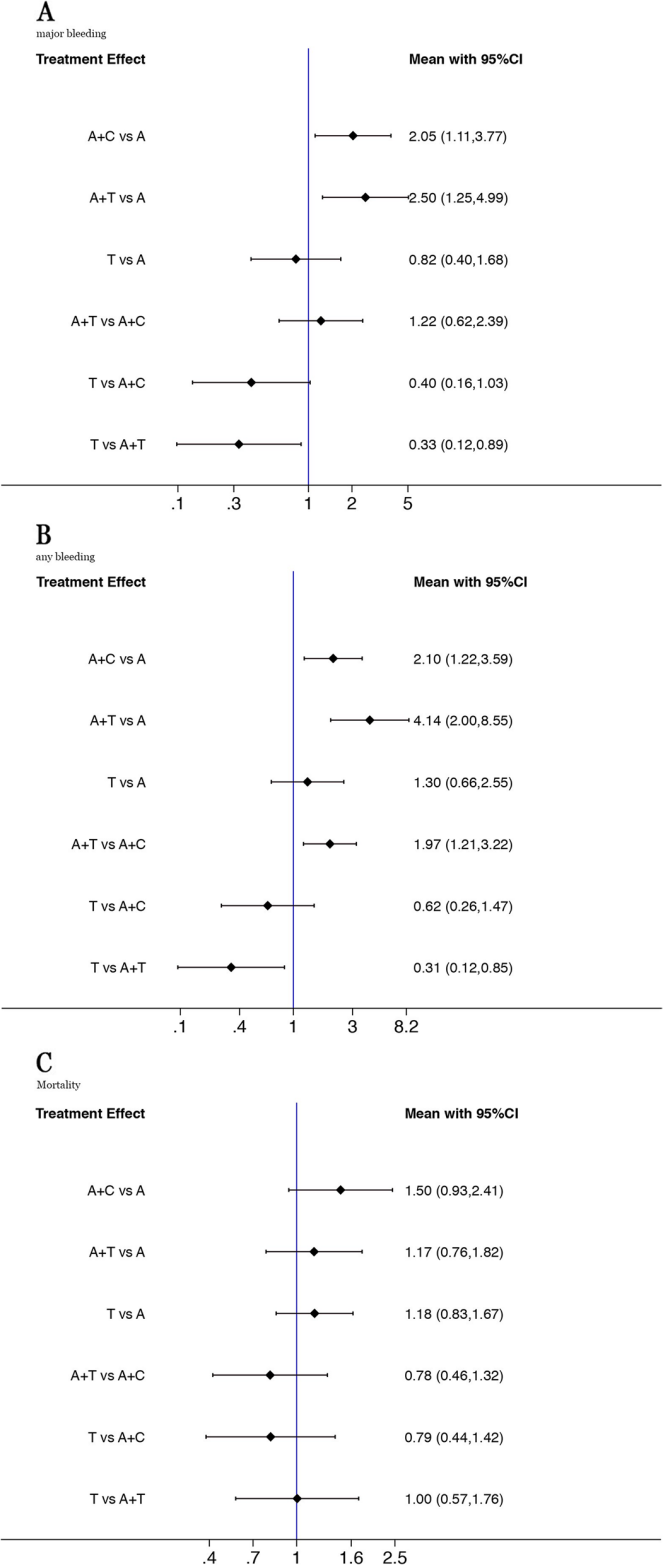
Forest plot for the safety outcomes between treatment vs control (**A**: major bleeding, **B**: any bleeding, **C**: mortality)

### Ischemic stroke

All the 7 RCTs with 41,745 participants reported the ischemic stroke event. There was no significant difference between the two DAPT regimens in preventing ischemic stroke recurrence(OR, 1.16; 95% CI, 0.93–1.45). The two DAPT regimens were both more efficacious compared with aspirin alone (clopidogrel: OR, 0.68; 95% CI, 0.58–0.78, ticagrelor: OR, 0.66; 95% CI, 0.49–0.88)) (Fig. 2B).SUCRA values suggested that clopidogrel and aspirin may be associated with the lowest risk of recurrent ischemic stroke (SUCRA,96.5%), then ticagrelor and aspirin (SUCRA,80.8%), and ticagrelor and aspirin alone (SUCRA, 39.0% and 0.9%) (Figure S3B).

### Composite of vascular events (stroke, myocardial infarction, and vascular death)

6 RCTs with 30,729 participants reported composite of vascular events.There was no significant difference between the three antiplatelet drugs for preventing composite of vascular events (Fig. 2C).Ticagrelor and aspirin had the highest SUCRA value at 80.8%, followed by ticagrelor (SUCRA, 51.4%) (Figure S3C).

### Major bleeding

All the 7 RCTs reported tth major bleeding. DAPT with clopidogrel-aspirin or ticagrelor-aspirin both increased the major bleeding compared with aspirin alone (clopidogrel: OR, 2.05; 95% CI, 1.22- 3.77; ticagrelor: OR, 2.55; 95% CI, 1.25–4.99). there was no significant difference in the number of major bleeding between the two DAPT regimen (OR, 1.22; 95% CI, 0.62,2.39). Ticagrelor did not increase the risk of major bleeding compared with aspirin (OR, 0.82; 95% CI, 0.40,1.68) (Fig. 3A). All were low evidence recommendations. SUCRA values further suggested that mono antiplatelet therapy was associated with the lowest risk of major hemorrhage (SUCRA, aspirin76.1%, Ticagrelor88.6%), followed by clopidogrel and aspirin (SUCRA,25.4%) and ticagrelor and aspirin (SUCRA,9.9%) (Figure S3D).

### Any bleeding

4 RCTs with 25,848 participants reported any bleeding.DAPT with Clopidogrel or ticagrelor and aspirin both increased any bleeding compared with aspirin alone (clopidogrel: OR, 2.10; 95% CI, 1.22- 3.59; ticagrelor: OR, 4.14; 95% CI, 2.00–8.55). Ticagrelor and aspirin did increase the risk of any bleeding compared with clopidogrel and aspirin (OR, 1.97; 95% CI, 1.21,3.22) (Fig. 3B). SUCRA values further suggested that ticagrelor and aspirin was associated with the highest risk of any bleeding (SUCRA, 0.4%). (SUCRA, 92.7%), followed by ticagrelor(SUCRA,68.9%),clopidogrel and aspirin (SUCRA,38.0%) and (Figure S3E).

### Mortality

6 RCTs with 41,353 participants reported mortality events.There was no difference in the risk of mortality between any of the treatment regimens (Fig. 3C). However, SUCRA values could provide some indication that aspirin alone was associated with the highest SUCRA value (84.2%) for the lowest mortality, and clopidogrel and aspirin was associated with the highest mortality (SUCRA14.3%) (Figure S3F).

### Subgroup analysis

Subgroup analysis was conducted among four RCTs with 16,115 Asian participants mainly Chinese for ischemic stroke events and 16,085 for major bleeding events. Subgroup analysis showed that ticagrelor and aspirin reduced the risk of ischemic stroke recurrence (OR, 0.77; 95% CI, 0.63- 0.92)without increasing the risk of major bleeding (OR 0.94; 95% CI 0.45–1.95) in the Asian population, compared with clopidogrel and aspirin (Table S1).

### Sensitivity analysis

Sensitivity analysis with excluding CHANCE-2 remained unchanged for ischemic stroke and major bleeding (Table 2).

### Inconsistency and publication bias

**Fig. 4:**
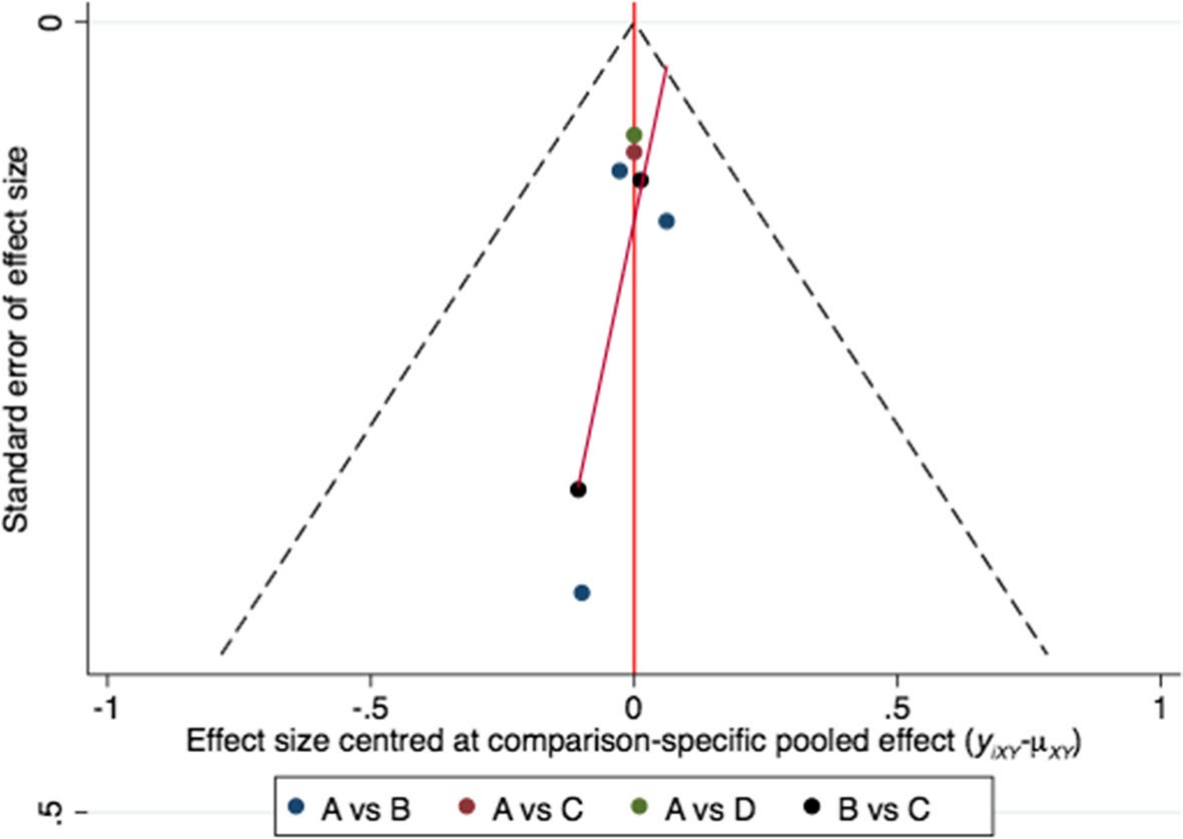
Funnel plot did not show significant publication bias in this study

There was inconstancy in stroke recurrence and ischemic stroke recurrence between the direct and indirect evidence, node-splitting method indicated local inconsistency. The inconstancy model was adoted to statistical processing. There was no inconstancy in the safety events.The results of the funnel plot did not reveal significant publication bias in stroke recurrence endpoint (Fig. 4).

## Discussion

This systematic review and NMA indicated that there was no significant difference between the two DAPT regimens in terms of stroke recurrence, ischemic stroke,and the composite of vascular events and mortality. Either regimen of DAPT was superior to aspirin alone for preventing stroke and ischemic stroke recurrence. While both DAPT regimens were associated with higher rates of major bleeding and any bleeding compared with aspirin alone, no difference was noted between the two DAPT regimens in major bleeding. But ticagrelor and aspirin mainly owing to mild bleeding.

Based on the SUCRA results, it was obvious that clopidogrel and aspirin seemed to be the most preferable interventions in terms of stroke and ischemic stroke endpoints and ticagrelor and aspirin for the composite of vascular events. While ticagrelor plus aspirin was associated with the highest risk of major bleeding and any bleeding.

The conclusion of our NMA is consistent with previous meta analysis [17, 18] and RCTs [5, 6, 14], which recommended DAPT for secondary prevention in high-risk Patients with new TIA and minor ischemic stroke. We updated the previous meta analysis including the head to head RCT with a direct comparison between the two DAPT regimens.

As we all know, ticagrelor does not require metabolic activation for its antiplatelet effect, and may yield greater levels of inhibition of platelet aggregation than clopidogrel in acute coronary syndromes and minor stroke [23, 24], however,we did not find that ticagrelor plus aspirin was superior to clopidogrel plus aspirin for the secondary prevention in stroke, these can be interpreted carefully as explained below.

Firstly, the genetic background of enrolled patients can potentially confound the interpretation. The prevalence of clopidogrel resistance in the general population showed different geographic patterns and ethnicity [11]. The loss-of-function allele for the CYP2C19 gene was approximately 30% of the US population and 60% in Chinese populations, clopidogrel plus aspirin reduced the risk of a new stroke only in the subgroup of patients who were not carriers of the CYP2C19 loss-of-function alleles [25]. Ticagrelor was superior to clopidogrel in inhibiting platelet reactivity among patients with acute minor stroke or TIA who were carriers of the CYP2C19 LOF alleles [24]. Our NMA only contained about 50% Asian population. our subgroup analysis indicated that ticagrelor and aspirin reduced the risk of ischemic stroke recurrence and did not increase the risk of major bleeding in the Asian population compared with clopidogrel and aspirin. CHANCE-2 clinical trial including individuals mainly carriers of the CYP2C19 loss-of-function alleles, ticagrelor and aspirin was superior to clopidogrel and aspirin in reducing the risk of subsequent stroke and did not increase the risk of severe or moderate bleeding. These implied that the population carriers of the CYP2C19 LOF alleles will benefit from therapy with ticagrelor.

Secondly, patients with large-artery atherosclerosis after a TIA or minor stroke have a higher risk of stroke recurrence among ischemic stroke etiologic subtypes [26]. THALES-atherosclerosis subgroup showed that ticagrelor and aspirin versus aspirin provided a clinically meaningful benefit [27]. Stroke recurrence was markedly lower in the ticagrelor and aspirin group than in the clopidogrel and aspirin group in PRINCE large artery atherosclerosis population [15]. We also noticed that ticagrelor and aspirin had the highest SUCRA value in the composite of vascular events which included cardiovascular events, Previous acute coronary syndrome study confirmed that ticagrelor as compared with clopidogrel significantly reduced the rate of death from vascular causes, myocardial infarction, or stroke without an increase in the rate of overall major bleeding [12]. These implied a hypothesis that ticagrelor may benefit those in higher risk of ischemic populations and cardiovascular events.

Finally, the major concern of ticagrelor plus aspirin is the higher risk of bleeding events. Compared with clopidogrel, patients on ticagrelor are at a twofold increased risk for a recurrent stroke or TIA, a twofold increased risk for an intracranial hemorrhage complication, and a tenfold increased risk for a fatal intracranial hemorrhage in acute coronary syndrome patients with a history of cerebrovascular disease [28, 29]. This difference could be partly due to the long term DAPT use, and partly due to the higher risk of hemorrhagic stroke in cerebrovascular disease. Severe bleeding occurred in 0.5% of patients with ticagrelor and aspirin in THALES [14] and 0.3% in CHANCE-2 [16], and CHANCE-2 showed a similar trend of the increased rate of any bleeding events, but not in severe hemorrhagic risk. Because the population included in CHANCE-2 were in CYP2C19 Loss-of-Function Carriers, who were at a higher risk of ischemia stroke and relatively lower risk of bleeding after treatment failure with clopidogrel plus aspirin. This may imply that ticagrelor and aspirin mainly benefit patients at higher risk for recurrent ischemia but lower risk for hemorrhage.

Although our NMA did not find a superiority of ticagrelor plus aspirin, it was at least as effective and safe as aspirin plus clopidogrel in stroke prevention, patients with CYP2C19 Loss-of-Function Carriers [16], large-artery atherosclerosis [15] and other higher risk for recurrent ischemia but lower risk for hemorrhage may benefit from the ticagrelor plus aspirin. CYP2C19 genotyping to guide antiplatelet therapy for acute minor strokes and high‐risk TIAs was highly cost‐effective in China [30], although routine genetic testing for clopidogrel resistance was not currently recommended for any indication.

Our NMA had several limitations. Firstly, the studies included varied in terms of the study sample, treatment and follow-up duration, and the outcome was only reported in THALES trial up to 30 days. while our statistical analysis strategy using actually follows patients for the entire 90-day period. Only the CHANCE-2 trial was in CYP2C19 Loss-of-Function Carriers. which may result in a random error. Secondly, only published data were included, which may cause potential publication bias. Visible funnel plot was limited in an NMA owing to the limited number of studies for each pairwise comparison. Thirdly, different criteria for endpoint events and design were adopted. which may result in potential heterogeneity and influence evaluation. Although these were minimal.

In summary, there was no statistically significant difference between clopidogrel plus aspirin and ticagrelor plus aspirin and DAPT was superior to aspirin in the prevention of stroke, although ticagrelor plus aspirin was a higher risk of any bleeding, our NMA suggested aspirin and ticagrelor will be a reasonable alternative to aspirin and clopidogrel in patients who treatment failure with clopidogrel plus aspirin. because of the low evidence recommendations, further head-to-head RCTs in populations with large-artery atherosclerosis and other higher risk for recurrent ischemia but lower risk for hemorrhage will be needed to confirm.

### Supplementary Information

**Additional file 1: Figure S1.** Flow chart of included/excluded studies

**Additional file 2: Figure S2.** The risk of bias of individual studies using the RoB2

**Additional file 3: Figure S3.** Plots of the surface under the cumulative ranking curves for all treatments in different events (Fig. S3A: stroke, Fig. S3B: ischemic stroke, Fig. S3C: vascular events, Fig. S3D: major bleeding, Fig. S3E: any bleeding, Fig. S3F: mortality)

**Additional file 4: Table S1.** Efficacy and Safety outcome with Available Data in Asian subgroup

## Data Availability

The authors confirm that the data sets analyzed in the present study are available from the corresponding author upon reasonable request.
