# Supplementary material for: Ticagrelor plus aspirin in patients with minor ischemic stroke and transient ischemic attack: a network meta-analysis
Source: BMC Neurol. 2023 Aug 14;23:303. doi: 10.1186/s12883-023-03356-7 (PMC10424353; doi:10.1186/s12883-023-03356-7)
Supplement: Supplementary file 1 — Additional file 1: Figure S1. Flow chart of included/excluded studies [file 12883_2023_3356_MOESM1_ESM.docx]

53 only published abstract

21 protocols

2910 irrelevant

70meta analysis and reviews

39subgroup

6 subgroup

7 editorial

14 non-RCT

20 population or control

5 meta-analysis and reviews

1pilot study

7 Studies included in the quantitative synthesis

3153 records screened on basis of titles and abstracts

60 Full text articles assessed for eligibility

1 records from other reviews

3152records identified through database searching

Figure S1 Flow chart of included/excluded studies
