# Supplementary figures and images for: Ticagrelor plus aspirin in patients with minor ischemic stroke and transient ischemic attack: a network meta-analysis
Source: BMC Neurol. 2023 Aug 14;23:303. doi: 10.1186/s12883-023-03356-7 (PMC10424353; doi:10.1186/s12883-023-03356-7)

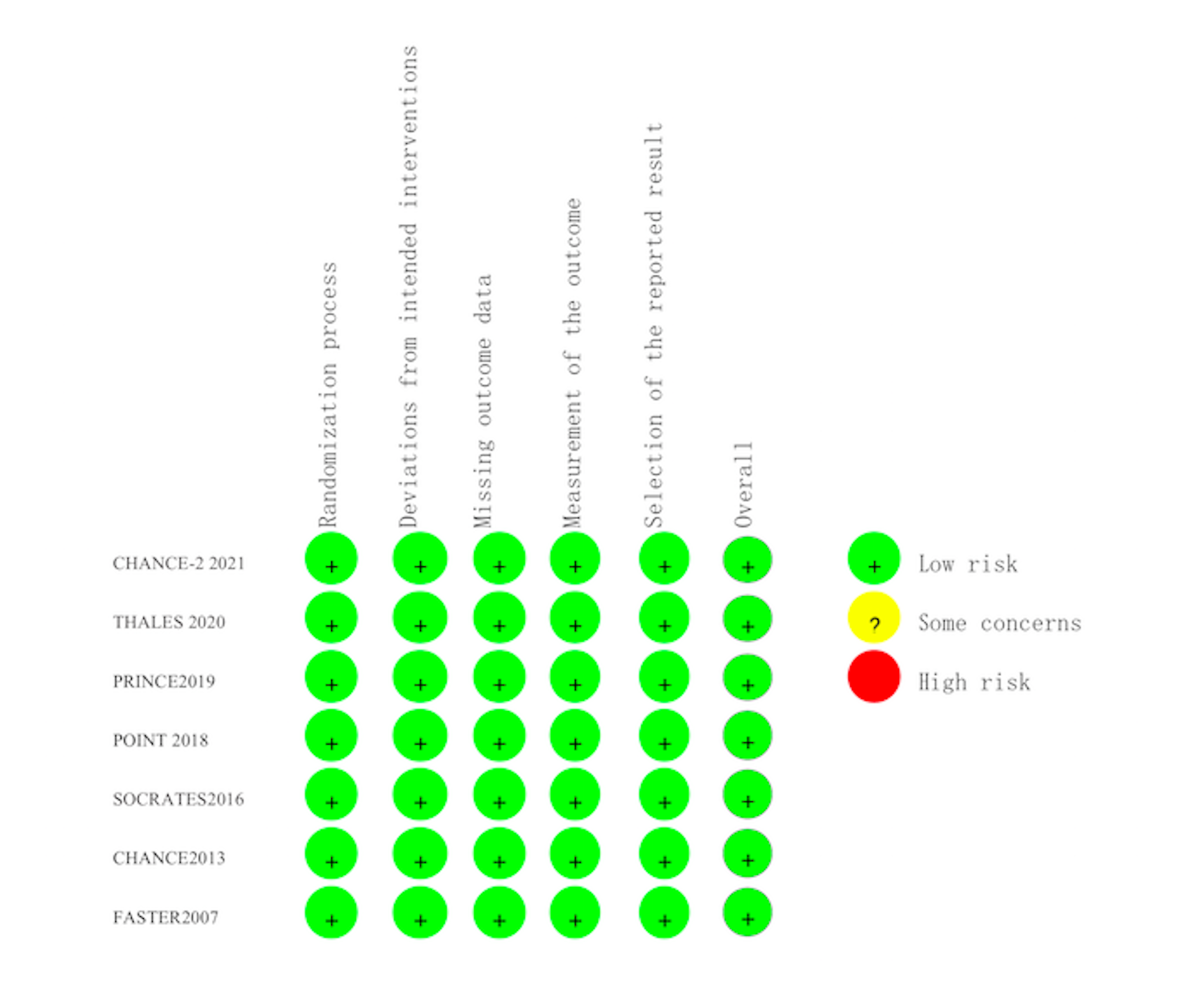


Figure S2 The risk of bias of individual studies using the RoB2

Supplement: Supplementary file 2 — Additional file 2: Figure S2. The risk of bias of individual studies using the RoB2 [file 12883_2023_3356_MOESM2_ESM.docx]
