# Supplementary material for: Ticagrelor plus aspirin in patients with minor ischemic stroke and transient ischemic attack: a network meta-analysis
Source: BMC Neurol. 2023 Aug 14;23:303. doi: 10.1186/s12883-023-03356-7 (PMC10424353; doi:10.1186/s12883-023-03356-7)
Supplement: Supplementary file 3 — Additional file 3: Figure S3. Plots of the surface under the cumulative ranking curves for all treatments in different events (Fig. S3A: stroke, Fig. S3B: ischemic stroke, Fig. S3C: vascular events, Fig. S3D: major bleeding, Fig. S3E: any bleeding, Fig. S3F: mortality) [file 12883_2023_3356_MOESM3_ESM.docx]

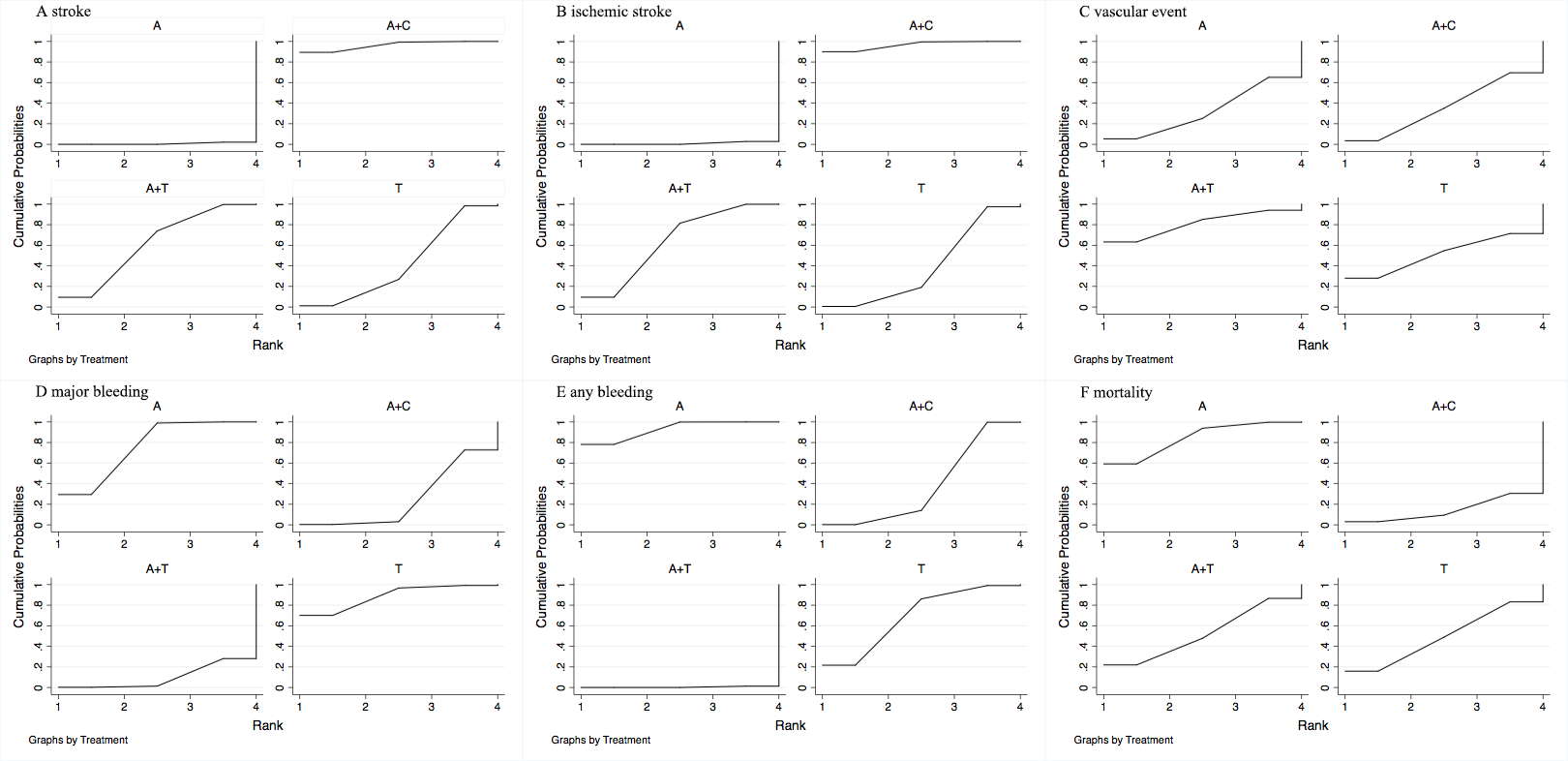


**Figure S3** Plots of the surface under the cumulative ranking curves for all treatments in different events(FigureS3A: stroke, FigureS3B:ischemic stroke , FigureS3C: vascular events, FigureS3D: major bleeding, FigureS3E:any bleeding , FigureS3F: mortality)
