# Supplementary material for: Ticagrelor plus aspirin in patients with minor ischemic stroke and transient ischemic attack: a network meta-analysis
Source: BMC Neurol. 2023 Aug 14;23:303. doi: 10.1186/s12883-023-03356-7 (PMC10424353; doi:10.1186/s12883-023-03356-7)
Supplement: Supplementary file 4 — Additional file 4: Table S1. Efficacy and Safety outcome with Available Data in Asian subgroup [file 12883_2023_3356_MOESM4_ESM.docx]

| Outcome measure | OR (95% CI) | | |
| --- | --- | --- | --- |
|  | A+C vs A | A+T vs A | A+T vs A+C |
| ischemic stroke | 0.67 (0.55,0.80) | 0.51 (0.39,0.66) | 0.77 (0.63,0.92) |
| major bleeding | 0.88 (0.32,2.42) | 0.82 (0.23,2.87) | 0.94 (0.45,1.95) |

A, aspirin; C, clopidogrel;T, ticagrelor

Table S1. Efficacy and Safety outcome with Available Data in Asian subgroup.
